# Supplementary material for: A prospective cohort study to describe the morphology of buboes in patients with bubonic plague using ultrasound imaging
Source: PLoS Negl Trop Dis. 2026 Mar 16;20(3):e0014133. doi: 10.1371/journal.pntd.0014133 (PMC13004500; doi:10.1371/journal.pntd.0014133)
Supplement: Attachment — Submitted filename: Response to reviewers_Round 2.pdf [file pntd.0014133.s003.pdf]

Dear Editorial team,

We are grateful to both reviewers for the time and expertise they invested in evaluating our manuscript.

With regard to the comments from Reviewer 1, we have responded carefully and substantively to all scientific critiques in both rounds of the peer review process. However, we would respectfully note that several comments were framed in a manner that we found dismissive of the study's context and constraints, and at times extended beyond methodological critique to broader judgments about the study's value. While we fully welcome rigorous scientific scrutiny, we believe that peer review is most effective when critiques are proportionate, acknowledge the study's context and are expressed in a constructive and collegial manner.

We leave it to the editorial team to provide the final judgement on our manuscript based on our responses in both rounds of review, but we wished to bring this to your attention in the interest of maintaining a fair and professional review process.

Please find below our responses:

Reviewer feedback	Author response
We note that your Data Availability Statement is currently as follows: "Aggregated data is available and openly accessible via the following link: https://projects.vertex.isaric.org/?param=Bubo_Images. The protocol is available on the ISARIC website: https://isaric.org.". Please confirm at this time whether or not your submission contains all raw data required to replicate the results of your study. Authors must share the "minimal data set" for their submission. PLOS defines the minimal data set to consist of the data required to replicate all study findings reported in the article, as well as related metadata and methods (https://journals.plos.org/plosone/s/data-availability#loc-minimal-data-set-definition).	Yes, the repository contains the raw data required to replicate the results.
Please amend your detailed Financial Disclosure statement. This is published with the article. It must therefore be completed in full sentences and contain	We have amended the financial disclosure statement.

the exact wording you wish to be published	
The images are blur as PDF. Should show better.	We have re-uploaded the figures in higher resolution.
The absence of any control group, either apparently healthy matched people / either patients with other acute infection, is just rendering impossible to reach goals of the study.	We appreciate the reviewer's comments, but we would like to emphasise that our study was conducted under challenging conditions that inevitably influenced recruitment and sample size. The study was implemented in rural health centres in Madagascar, where the geographic distribution of plague "hotspots" is both unpredictable and variable from season to season. We selected sites with a consistent history of reported cases; however, during the study period, overall case numbers were lower than anticipated, due to natural fluctuations in annual transmission patterns. In addition, funding constraints limited both the number of participating sites and the duration of recruitment. As a result, we enrolled fewer participants than originally projected, including four individuals who were ultimately classified as non-cases.<br><br>We fully acknowledge that the modest sample size (particularly the small number of non-cases) limits the scope of inference that can be drawn. For this reason, we have intentionally focused our analysis on the confirmed case cohort and present the findings in a primarily descriptive manner. We believe this approach is transparent and appropriate given the study context.<br><br>However, we respectfully disagree with the assertion that the study offers no new insights into plague. First, little has been previously published about bubo morphology and evolution over time. Our study provides systematically collected clinical and radiological data on these features within a defined patient cohort; data that, to our knowledge, have not previously been reported in a structured,

	longitudinal manner. Second, our findings directly challenge the prevailing assumption that bubo size and morphology can reliably serve as indicators of treatment response. These parameters are routinely reported in case descriptions and have been incorporated as endpoints in clinical trials. By demonstrating variability and limited reliability in these measures within confirmed cases, our study highlights an important gap between clinical practice and empirical evidence.<br>In this respect, the study contributes novel and practically relevant data generated under real-world conditions in an endemic setting. We believe these findings are important for clinicians, researchers, and trial methodologists, as they suggest that bubo size and morphology should be interpreted with caution when used as markers of treatment response. Even within the constraints of a small cohort, this observation provides valuable information that can inform future research design and clinical assessment strategies.
The on-going affirmation of “normal buboes” is just non-sense.	In our manuscript we do not refer to “normal buboes”. The only place we have found a similar reference is in the caption in Figure 1 where we present a bubo with “normal features”. We have changed this wording to “without abnormal radiological findings”.<br><br>Where the term “normal” appears in other parts of the manuscript, it refers specifically to defined radiological characteristics of lymph nodes that lack features typically associated with pathological involvement. In these instances, “normal” denotes the absence of abnormal radiological findings according to established imaging criteria (e.g., preserved morphology, absence of necrosis, no surrounding inflammatory

	changes), rather than implying clinical normality in a broader sense.
The still discrepancy in data, illustrated by contradictory line 20 / line 29 in the abstract	While the specific discrepancy is not detailed in the reviewer's comment, we presume it refers to line 20, which states that the study concerns confirmed bubonic cases, and line 29, which indicates that suspected cases were enrolled, some of whom were subsequently confirmed.<br><br>We would like to clarify that there is no inconsistency. The study aim was to characterise confirmed bubonic plague cases; however, as is standard in prospective clinical research conducted in endemic settings, patients were enrolled at presentation on the basis of suspected disease and were subsequently classified according to confirmatory testing. The abstract reflects this process: it states the overarching aim (confirmed cases) while accurately describing the enrolled population (suspected cases, of whom a subset were confirmed).<br><br>We believe the abstract appropriately summarises both the study objective and the patient population. Full methodological details, including case definitions and confirmation procedures, are provided in the Methods section.
The absence of profound lymph nodes ultrasonographic examination, where ultrasonography would be most valuable: in fact, in the study, clinics (palpation n = 12) was superior to ultrasonography (n= 10)!	We are unsure which part of the manuscript this comment relates to. We would like to highlight however that the purpose of the study was to characterise the nodes, rather than to detect them. We believe this is represented clearly in the manuscript results and figures.
