# Supplementary material for: A prospective cohort study to describe the morphology of buboes in patients with bubonic plague using ultrasound imaging
Source: PLoS Negl Trop Dis. 2026 Mar 16;20(3):e0014133. doi: 10.1371/journal.pntd.0014133 (PMC13004500; doi:10.1371/journal.pntd.0014133)
Supplement: Attachment — Submitted filename: Response to reviewers.docx [file pntd.0014133.s002.docx]

**Response to reviewers**

Dear reviewers,

Thank you very much for providing detailed feedback on our articles. We have made several corrections to address the comments received during the peer-review process. The table below summarises the changes that have been made.

| **Reviewer feedback** | **Author response** |
| --- | --- |
| Please upload all main figures as separate Figure files in .tif or .eps format. For more information about how to convert and format your figure files please see our guidelines:  https://journals.plos.org/plosntds/s/figures | We have converted and uploaded all files in .tif format. |
| In the online submission form, you indicated that your data will be submitted to a repository upon acceptance. We strongly recommend all authors deposit their data before acceptance, as the process can be lengthy and hold up publication timelines. Please note that, though access restrictions are acceptable now, your entire minimal dataset will need to be made freely accessible if your manuscript is accepted for publication. This policy applies to all data except where public deposition would breach compliance with the protocol approved by your research ethics board. If you are unable to adhere to our open data policy, please kindly revise your statement to explain your reasoning and we will seek the editor's input on an exemption | The data has been added to our repository and publicly accessible via the link included in the Data Sharing section at the end of the manuscript. |
| Please amend your detailed Financial Disclosure statement. This is published with the article. It must therefore be completed in full sentences and contain the exact wording you wish to be published.  1) State the initials, alongside each funding source, of each author to receive each grant. For example: "This work was supported by the National Institutes of Health (####### to AM; ###### to CJ) and the National Science Foundation (###### to AM)." 2) State what role the funders took in the study. If the funders had no role in your study, please state: "The funders had no role in study design, data collection and analysis, decision to publish, or preparation of the manuscript." 3) If any authors received a salary from any of your funders, please state which authors and which funders. | We have amended the funding statement in the submission system as per these instructions. |
| Please send a completed 'Competing Interests' statement, including any COIs declared by your co-authors. If you have no competing interests to declare, please state "The authors have declared that no competing interests exist". Otherwise please declare all competing interests beginning with the statement "I have read the journal's policy and the authors of this manuscript have the following competing interests" | We have included the conflict of interest statement at the end of the manuscript. |
| To shorten, especially the Discussion ; as well as the « two cases » report. | We have modified the discussion section to be more succinct and concise. |
| To focus the goal : It could have been the neosemiology of plague, based on ultrasound imaging. | As outlined in the introduction, our study aimed to understand the relationship between bubo size, bubo morphology, and overall clinical status, as two recent clinical trials have used these measures as indicators of treatment response, and several other studies routinely report bubo size in their findings. Our objective was therefore to determine whether these measures meaningfully reflect disease progression and should continue to be considered clinically or operationally relevant endpoints. |
| Clearly report the form of X parameters used to remotely analyse data. In this perspective, the reviewer is interested in the number of ultrasoundly detected lymph nodes : only one, as generally reported in text books ? | We have more clearly stated the evaluated parameters to the methods section. |
| Compared these parameters between confirmed cases (i.e. true plague, with a direct demonstration of Y. pestis in any accurate clinical sample, including buboes !) with that of excluded cases. Doing so, authors could calculate the predictive values (both positive and negative) of this ultrasound sign, plus this one, plus this one | We have avoided inferential statistics as the number of enrolled patients is too small. |
| Abstract : clarify here (and in the text) whether the study was blind or not, for radiologists ? | We have specified in the abstract and methods that neither clinicians nor radiologists were blinded in the study. |
| « Normal » morphology for buboes is a hard concept (as well as in the text). | We included the diagram because normal and abnormal lymph node morphology can be difficult to interpret without visual reference. We felt that the figure would help readers understand the parameters we evaluated and this would improve the clarity of the manuscript. |
| Give topography for buboes. | The distribution of the buboes is already indicated on lines 187 and 188. |
| Clarify that only one diseased lymph node was detected, in how many patients, in how many anatomical sites ? | We have already stated on line 186 “At baseline, all participants presented with a single enlarged lymph node” and on line 201 we have stated that at D11 “two additional buboes detected in a single case”. |
| Authors summary is definitely too long (Yersinia pestis, lien 41). | We have revised the Author Summary to 192 words. |
| Line 75 : The reviewer argues that enteric form is nowadays a prevalent form ; and that in fact, deadly septicemia could follow iny one of the three forms (bubonic, enteric, pulmonary) | There is no evidence either in published literature or from cases seen in Madagascar (where approximately 80% of global cases are reported) to suggest that enteric plague is the most prevalent form. Current statistics show that bubonic plague is the most prevalent form, representing over 90% of annual cases. |
| Line 89 : The « s » indicates that several diseased lymph nodes could be detected in one anatomical site ibn one patient, a KEY data completely omitted in the present report. It is possible that this parameter is of interest for the differential ultrasoud diagnosis of buboe ; eventually usefull for the follow-up ( ??) in treated patients. Authors HAVE to go back to data to include this parameter in reading form and compare really confirmed patients (not the WHO criteria, medicine !) with firmly excluded patients | We have included an explanation in the methods about the reason we decided to follow just the index lymph node over time: “We measured only the index lymph node, as is standard in clinical practice and clinical trials, where the largest or most representative node is selected for follow-up over time.” |
| Table 1 adding nothing, has to be deleted | Table 1 gives a clear visual summary of the patient population. We will retain it in the manuscript. |
| Lines 191-192 : Figures unclear : how is-it possible to have 10 buboes for 12 cases, when buboes in the portal of entry ? | All participants presented with a bubo that was either detectable by physical examination or ultrasound. We evaluated the visibility of the bubo following physical examination. It is correct that while 12 confirmed cases of plague had a bubo, only 10 of those were visible on physical examination (without the assistance of imaging). |
| Lines 193-196 : Give figures for firmly diagnosed and firmly excluded cases. Which treatment ? What about excluded cases ? | This information is described on line 180-181. None of the screened patients were excluded. |
| Line 203 : i. Was laterality (right / left hand using) noted ? If so, did laterality correlate with that of buboes (especially for the upper body) ? | We did not record whether the patient was right or left handed. |
| Ultrasound machine used in the study should be described. | The ultrasound probe used in the study is already described on line 124. |
| Antibiotic treatment used in the cases should be stated. | We have added this information to the methods section. |
| Figures need improvement to appear better. I suggest changing the horizontal bar graphs comparing between day 1 and day 11 to vertical bars without Absent, making the comparison much easier. | We believe the figures represent the results of the study clearly and we have therefore not changed them. |
